# First hip hemiarthroplasty in a Göttingen Minipig; surgical and post-mortem protocol

**DOI:** 10.1186/s13018-024-05040-z

**Published:** 2024-09-06

**Authors:** Katrine Top Hartmann, Anders Odgaard, Ulrik Kragegaard Knudsen, Bent Aalbæk, Lasse Kvich, Julie Melsted Birch, Andreas Petersen, Thomas Bjarnsholt, Henrik Elvang Jensen, Louise Kruse Jensen

**Affiliations:** 1 Department of Veterinary- and Animal Sciences, University of Copenhagen, Grønnegårdsvej 7, 1870 Frederiksberg C, Denmark; 2 Department of Orthopedic Surgery, Copenhagen University Hospital, Rigshospitalet, Copenhagen, Denmark; 3 Department of Clinical Medicine, University of Copenhagen, Copenhagen, Denmark; 4 Costerton Biofilm Center, Department of Immunology and Microbiology, University of Copenhagen, Copenhagen, Denmark; 5 Center for Surgical Science, Department of Surgery, Zealand University Hospital, Køge, Denmark; 6 Statens Serum Institut, Artillerivej 5, 2300 Copenhagen S, Denmark; 7 Department of Clinical Microbiology, Copenhagen University Hospital, Rigshospitalet, Copenhagen, Denmark

**Keywords:** Prosthetic joint infection, Göttingen minipig, Animal model, Prosthesis, Biofilm

## Abstract

**Background:**

Prosthetic joint infections (PJI) are recalcitrant, hard-to-treat infections and severe complications of joint arthroplasty. Therefore, there is a need to develop new effective treatment strategies, and animal models of high clinical relevance are needed. This study aimed to develop a detailed surgical protocol for hip hemiarthroplasty in Göttingen minipigs and a thorough post-mortem sampling protocol to pave the way for creating a minipig PJI model.

**Methods:**

Three adult female Göttingen minipigs underwent surgery with insertion of a hip hemiarthroplasty, using the anterior approach to the hip joint. After surgery the minipigs were followed closely with daily clinical evaluation and gait scoring. Comprehensive post-mortem analyses were performed with evaluation of macroscopic lesions, microbiology, synovial fluid analysis and histology.

**Results:**

The study resulted in the first Göttingen minipig with hip hemiarthroplasty and identified several points of awareness when inserting a hip prosthesis in minipigs, especially the high risk of joint dislocation. A spontaneous PJI occurred in one of the minipigs, revealing an impaired ability of the immune cells to reach the bacteria at the bone-prosthesis interface.

**Conclusion:**

The present study provides a detailed description of surgical technique and post-mortem sampling and validates the suitability of the hip hemiarthroplasty minipig model for future experimental modeling of PJI.

**Supplementary Information:**

The online version contains supplementary material available at 10.1186/s13018-024-05040-z.

## Introduction

The risk of prosthetic joint infection (PJI) following total hip or knee arthroplasty is 1–2% [1–4]. However, with a globally increasing number of primary arthroplasties performed each year and an increasing population of elderly patients and patients with co-morbidities such as diabetes and immunosuppression, the annual number of PJIs is estimated to rise dramatically in the coming years [3, 5]. Alone in the United States 4 million hip and knee arthroplasties are predicted by 2030 and the annual financial burden of the associated PJIs is estimated to amount 1.85 billion dollars [4]. PJI poses severe complications for patients presenting as destabilization of the prosthesis functional impairment of the affected limb, pain, tissue necrosis and an impaired quality of life with psychosocial distress, isolation, depression, and anxiety, at levels comparable to those reported for cancer patients [4, 6]. Additionally PJIs are very hard-to-treat infections, requiring complex multidisciplinary management with prolonged antibiotic treatment and revision surgeries [6], which brings a serious financial burden to the healthcare systems, and challenges the global use of antimicrobials [6].

Therefore, gaining more knowledge about PJI development and finding and testing new diagnostic tools and treatment strategies is crucial. Large animal models that can imitate the clinical aspect of PJI are essential for this purpose [7, 8]. A PJI animal model should closely replicate the periprosthetic environment to ensure the highest clinical relevance. The prosthesis should be made of relevant materials, design, and geometry, separate the intraarticular space from the intramedullary space, and support load. Further, the model should be performed in animals with immunological and musculoskeletal properties comparable to humans, such as swine [7]. Previously pigs were often dismissed as experimental animals due to their rapid growth, large adult size, and need for specialized large animal housing facilities. However, the size and growth challenges have been overcome by the Göttingen minipigs, which reach a mature weight of 30–50 kg and skeletal maturity at approximately 18 months [9], which make them a viable choice for arthroplasty and PJI studies, which optimally should be based on adult animals.

The current study aimed to develop a surgical procedure for hip replacement in adult Göttingen minipigs and a comprehensive animal welfare and post-mortem evaluation protocol. The present study led to the first Göttingen minipig with a hip hemiarthroplasty, paving the way for future minipig PJI models of the highest clinical relevance. The present study is descriptive and, therefore, based on a limited number of animals which, however, provided pivotal experiences and suggestions for further best practice protocols.

## Methods

### Animals and housing

The experimental protocol was approved by The Danish Animal Experiments Inspectorate (license No. 2022-15-0201-01130). The study was conducted at the University of Copenhagen, Frederiksberg, Denmark. Three adult female Göttingen Minipigs (Ellegaard Göttingen Minipigs A/S, Dalmose, Denmark) were included. The minipigs (40–48 kg body weight) were 19–26 months old at study start. All minipigs had previously been used for breeding (1–3 litters). The minipigs were acclimatized for two weeks prior to start and barrier-housed in single pens with the possibility of snout-to-snout contact. The minipigs were fed twice daily with a commercial pig diet* (Brogaarden Altromin, 9069—Extrudate)*, had free access to tap water, and had a 12-h light/ dark cycle.

### Anesthesia, analgesia and euthanization protocol

**Fig. 1:**
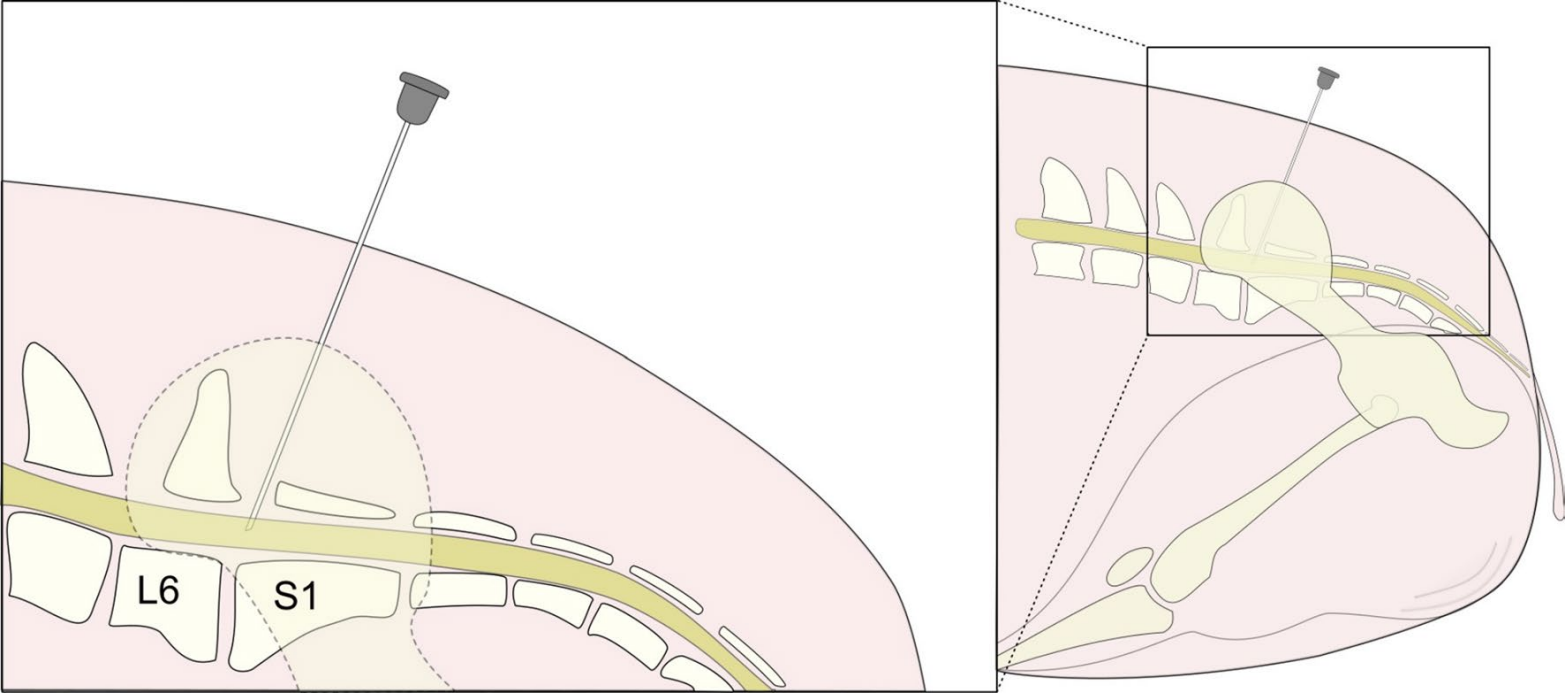
Needle placement and injection of epidural block on minipigs. An epidural block was injected into the epidural space between L6 and S1, with the minipig positioned in sternal recumbency with the hindlegs pulled forward to open the lumbosacral junction. The figure is inspired by Skarda, Roman T., [9]

Intramuscular sedation was followed by intravenous infusion of Propofol, and intraoperative analgesia was achieved by intravenous infusion of Fentanyl. Intra-operative and postoperative analgesia was achieved with an epidural block consisting of Morphine and Bupivacain sterilely injected into the epidural space between the lumbosacral junction of L6 and S1 (Fig. 1) [10]. The reported postoperative analgesic time for this procedure is 24 h in dogs [11]. Before surgery, the minipigs received an intramuscular injection of Meloxicam, providing postoperative analgesia for 24 h. Following surgery, the minipigs received daily oral analgetic treatment with Meloxicam. In case of lameness or observed pain behavior, an intramuscular injection of Buprenorphine was provided every eighth hour. The minipigs were euthanized by intravenous injection of an overdose of pentobarbital. See supplementary materials (Supplementary file 1) for complete protocols (time, dose, supplier).

### Prosthesis size, material, and design

Computed Tomography scanning and anatomical evaluations of five cadaveric adult female Gottingen minipigs revealed a uniformity of femur concerning size and geometry, allowing the same size and prosthesis design to be used in all animals. Cementless, titanium alloy (Ti-6AI-4V ELE (EBM)) Universal Hip Canine BFX^®^ Femoral Stem (BioMedtrix, New Jersey, USA) size 5 (designed for medium-sized dogs) was used together with a Cobalt Chrome Universal Hip Canine Femoral Head (BioMedtrix, New Jersey, USA) size 17 + 0 mm. A hemiarthroplasty was performed, i.e. no insertion of an acetabular cup component.

### Surgical procedure by anterior approach to the hip joint

All minipigs were placed in left lateral recumbency. Anatomical landmarks of the greater trochanter, hip, and knee joint were marked. The entire thigh area, from the knee to the spine, was clipped and aseptically prepared by washing using Medi-scrub (Medi-Skrub, Meda AS, Allerød, Denmark) and disinfection with chlorhexidine-ethanol (0.5% chlorhexidine, 85% ethanol). Surgical drapes were placed around the surgical area, and the area was covered with Ioban (3M Ioban 2, 3Mdenmark) to reduce the risk of contamination [12].

**Fig. 2:**
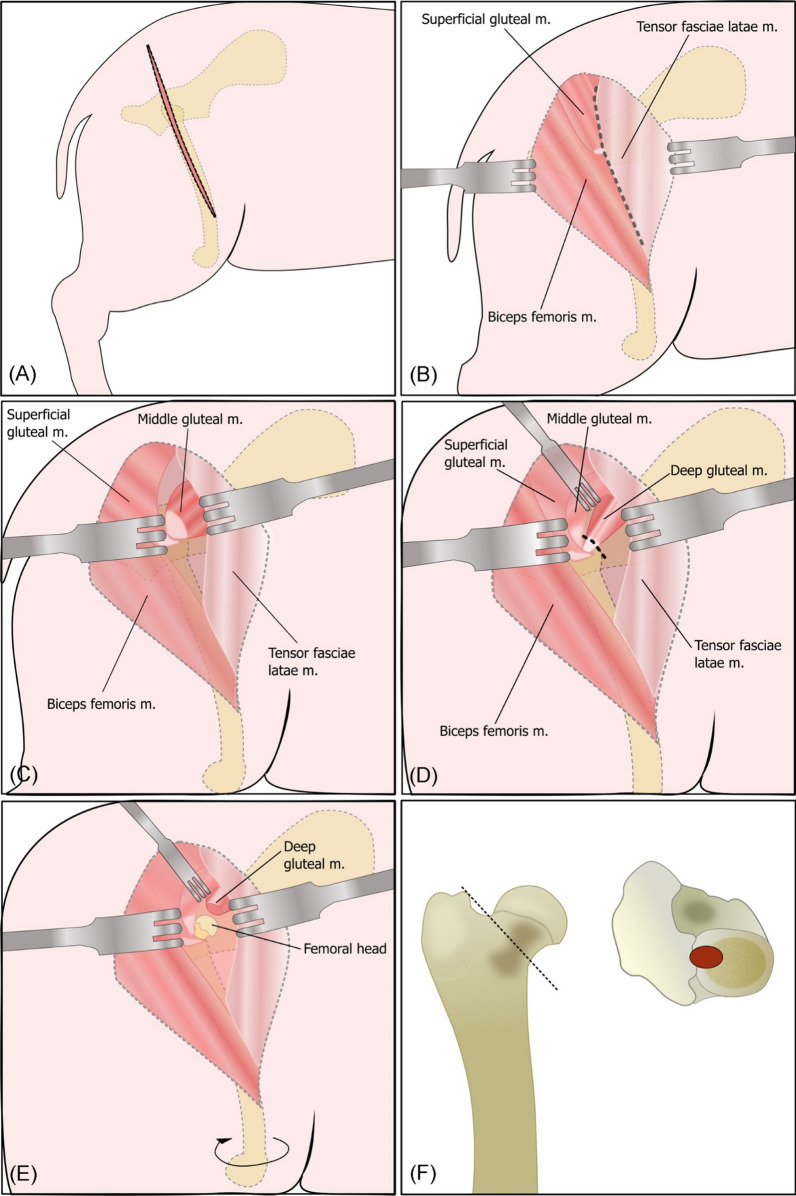
Anterior approach to the hip joint. The figure is inspired by Piermattei and Johnson, 2004 [12]. **a** Incision line. **b** Incision through the cleavage of superficial musculature. **c** Visualization of the middle gluteal muscle and its horizontal muscle fibers. **d** Visualization of deep gluteal muscle and incision at its attachment to the greater trochanter. **e** Incision of the joint capsule and external rotation of the femur allowing visualization of the femoral head. **f** Femoral neck resection at a 45° angle to the femoral bone (left). To open the femoral medullary cavity, a drill was used high on the resected neck (red mark, at the right). Figure F is inspired by Biomedtrix: Universal THR Surgical Technique *Pictorial Summary*

The anterior approach to the hip joint was applied by a modified version of the “Approach to the Craniodorsal Aspect of the Hip Joint Through a Craniolateral Incision” described by Piermattei and Johnson [13]. An incision was made through cutis and subcutis, starting two-thirds distally towards the knee, continuing towards the center of the greater trochanter, and ending halfway between the greater trochanter and the dorsal midline (Fig. 2a). The dense subcutaneous fat was undermined, and retractors were placed in the cutis and subcutis. A fasciae-incision was made in the cleavage along the cranial border of the biceps femoris muscle and the superficial gluteal muscle, and the caudal border of the tensor fasciae latae muscle (Fig. 2b). The biceps femoris muscle and superficial gluteal muscle were retracted caudally, whereas the tensor fasciae latae muscle was retracted cranially. The horizontal fibers of the middle gluteal muscle were now visible (Fig. 2c). By blunt dissection, the middle gluteal muscle was detached from the underlying muscles and retracted dorsally. The deep gluteal muscle and its attachments to the greater trochanter were now visible, and the joint space could be palpated underneath when rotating the femur externally. The deep gluteal muscle was cut near the greater trochanter and, by blunt dissection, detached from underlying tissue and retracted cranially (Fig. 2d). The cranial aspect of the joint capsule was now exposed, and a T-shaped incision was placed to open the joint. External femur rotation made the femoral head visible (Fig. 2e).

Caput femoris was elevated from the acetabular cup by a Hohmann retractor, and the femoral head ligament was cut by curved scissors. Femoral neck resection was performed close to the greater trochanter by an oscillating saw at a 45° angle (Fig. 2f). The proximal femur part was elevated to allow reaming. Prior to reaming, drilling (Drill 4 mm and 5 mm, BioMedtrix, New Jersey, USA) was used to access and enlarge the femoral medullary cavity (Fig. 2f). The femoral tapered reamer size four (BioMedtrix, New Jersey, USA) was used to widen the femoral medullary cavity prior to the impaction of broach size 4 followed by broach size 5 (BioMedtrix, New Jersey, USA). The femoral stem was inserted by press-fit attachment using the femoral impactor tool (Stem Impactor and Impactor Handle, BioMedtrix, New Jersey, USA) and gentle mallet strokes. The femoral head was attached to the femoral neck of the stem. Before the reduction of the joint, the acetabular cup was cleared up for remnants of the femoral head ligament. The surgical site was closed in layers, starting with the joint capsule, which needs to be closed thoroughly to add additional joint stability. Next, incised muscles were sutured, and subcutis and cutis were closed in separate layers. An antimicrobial ointment was applied to the surgical wound (Fucidin 2% Ointment, Leo Pharma, Ballerup, Denmark), followed by wound plast spray (Kruuse Wound Plast, Kruuse, Langeskov, Denmark).

### Radiographic evaluation

X-rays were obtained in lateral and ventro-dorsal positions using an X-ray system (Shimadzu Radspeed MC, Fuji, Tokyo, Japan) set to 79 kv and 18 mAs, respectively. The radiographs were taken during general anesthesia at the following time points: immediately after surgery (day 0) to ensure correct prosthesis placement, at day 14 post-surgery to give a midway study evaluation of the prosthesis, and at planned euthanasia. The X-rays were analyzed qualitatively to evaluate the positioning of the prosthesis within the acetabular cup, the alignment of the stem within the femur and the presence of any fractures.

### Postoperative care and clinical evaluation

The minipigs were monitored closely throughout the surgical recovery phase. Rubber mats were placed on the floor to ensure a non-slip surface. Initially, the minipigs were assisted with a blanket under the belly to minimize the load on the operated legs when trying to stand. When able to stand and walk without trembling, the blankets were removed. The minipigs were monitored several times a day following surgery and clinically evaluated once daily with a scoring of gait, wound, general status, activity level, and signs of pain-related behavior (Supplementary file 2). Impaired ability to stand, anorexia and systemic signs of infection, i.e., abnormal respiration or high fever, were set as humane endpoints.

### Macroscopic pathology

**Fig. 3:**
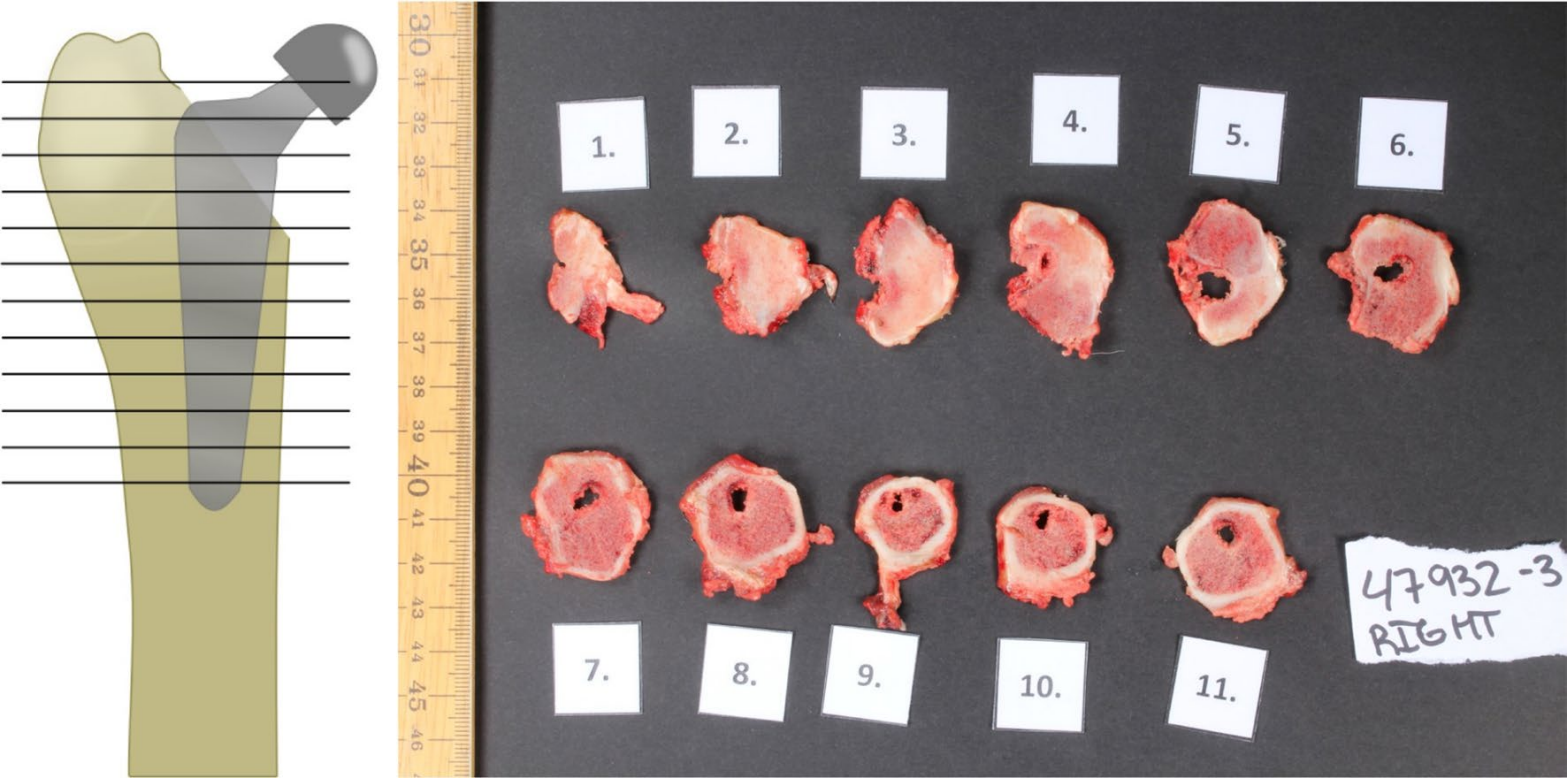
Post-mortem sectioning and sampling of the femoral bone. Horizontal sectioning of the femoral bone, after prosthesis removal, for bone evaluation and sampling for microbiology and histology

Following euthanasia, the minipigs were necropsied. The surgical wound was inspected and opened in layers, i.e., cutis, subcutis, and the different muscle layers using aseptic techniques. Tissue samples for histology were collected from each layer. The joint capsule was cut open using sterile instruments, and a sterile synovial fluid sample was collected using a syringe. The synovial membrane was collected for histology. The position of the artificial femoral head was evaluated, and the femur was removed together with the prosthesis. The prosthesis stability within the femoral bone was evaluated and followed by aseptic removal. The femoral bone was evaluated for signs of pus/abscess formation, necrosis, granulation tissue, fibrosis, and new bone formation. The proximal half of the femoral bone, which had formed the interface with the prosthesis, was cut into transverse sections of approximately 0.5 cm numbered from proximal to distal and collected for histological examination (Fig. 3). The left hip joint and femoral bone were opened, removed, and sampled correspondingly. The acetabulum from both hip joints was evaluated for gross lesions and sampled for histological evaluation. The major left and right deep inguinal lymph nodes were sampled for histology. The thorax and abdomen were opened, and all organs were inspected in situ. Samples from the liver, right kidney, and left caudal lung lobe were collected for histology.

### Histology

All tissue samples were fixed in 10% buffered formalin for five days. Following fixation, the bone samples were decalcified in a solution of 3.3% formaldehyde and 17% formic acid for six weeks. After fixation or decalcification, all samples were trimmed and processed through graded concentrations of alcohol and xylene, embedded in paraffin and sectioned (4–5 µm thickness). All sections were stained with Hematoxylin & Eosin (HE). On indication, bone sections were stained with immunohistochemistry (IHC) using antibodies towards staphylococci [14].

### Microbiology and sonication of prosthesis

Prior to surgery, nostril swabs, venous blood samples, and a cutis sample from the surgical field were collected for microbiological examination. Additionally, blood samples were collected prior to euthanization. During necropsy, tissue samples from the lung, cutis, subcutis, the biceps femoris muscle, the deep gluteal muscle, the synovial membrane, and three samples from the right femur, two from the proximal part of the prosthesis interface and one from the distal part of the prosthesis interface, were collected aseptically, using new sterile instruments for each sample. Furthermore, sutures from the subcutis, the profound muscle layer, and the joint capsule were collected for microbiological examination. Soft tissue, synovial fluid, blood, and swabs were inoculated on blood agar plates. Sutures were suspended in 100 µL sterile saline, of which 10 µL was inoculated on blood agar plates. All plates were incubated at 37 °C for 24 h under aerobic conditions. Morphologically distinct colonies were selected and identified by Matrix-assisted laser desorption ionization time-of-flight mass spectrometry (MALDI-TOF MS) (Vitek MS RUO, bioMérieux, Marcy-l'Etoile, France) [15]. Bone samples were aseptically homogenized and serially diluted in sterile isotone saline before 100 µl were plated on blood agar plates. The plates were incubated at 37 °C for 24 h under aerobic conditions. Morphologically distinct colonies were counted to estimate CFU/ml and identified with MALDI-TOF MS. Prostheses, collected aseptically at necropsy, were placed in 50 ml sterile centrifuge tubes and covered with sterile isotone saline. All prostheses were sonicated in an ultrasound bath for improved CFU counts [16, 17]. All microbiological evaluations were performed blinded. Whole genome sequencing was performed as previously described [18] on selected identified isolates to investigate relationship and origin.

### Synovial fluid

The color, viscosity and turbidity were evaluated, and two smears from each side were cytologically examined. If the cellularity was high enough, a differential count of leucocytes was carried out. The cytological examination of the synovial smears was carried out blinded.

### Statistics

Due to the small sample size of three minipigs, only descriptive analysis was carried out, without application of statistical tests or comparisons between the individuals.

## Results

### Insertion of prosthesis and postoperative radiographic evaluation

**Fig. 4:**
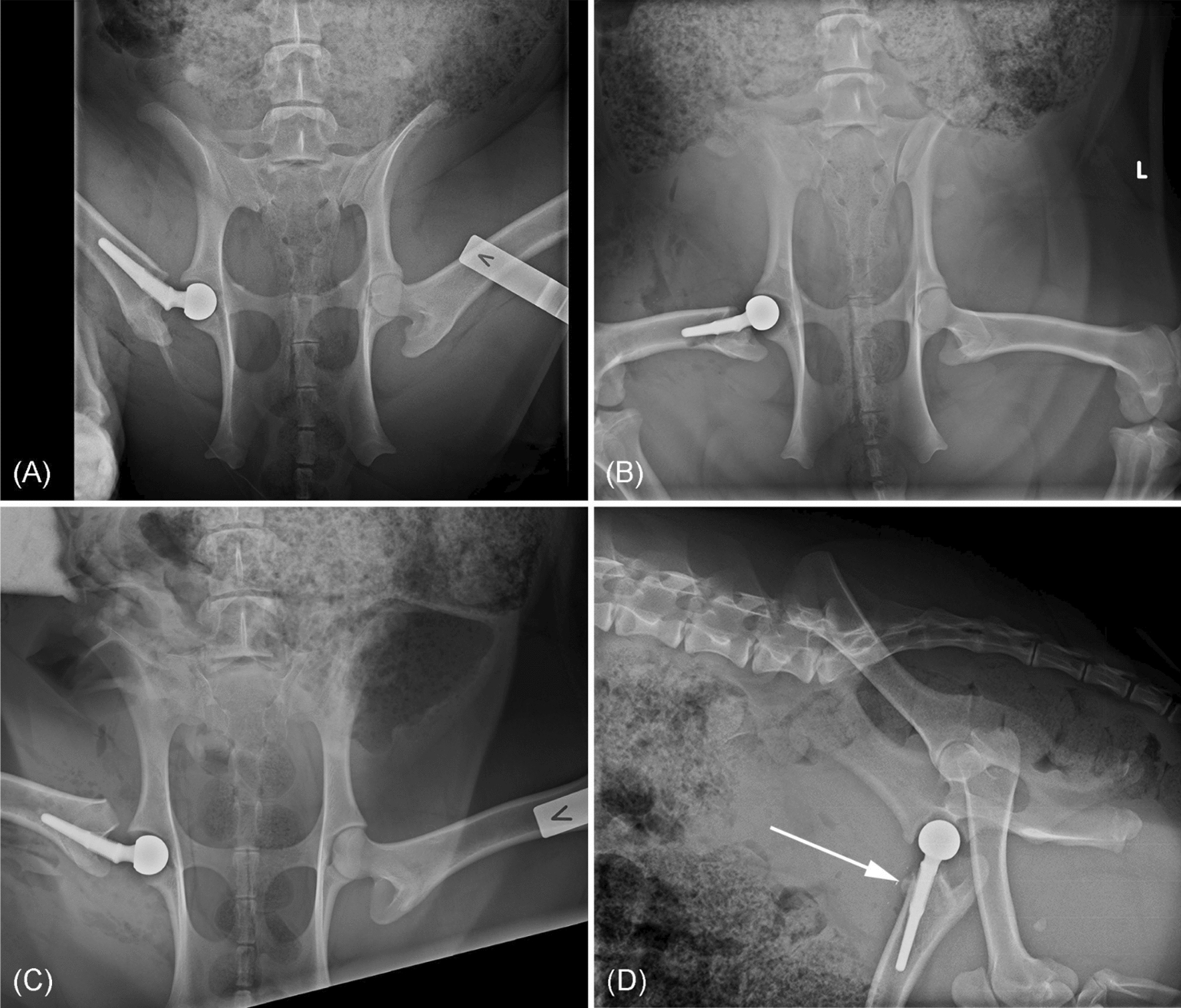
X-rays of the hip joint and femoral stem. Ventro-dorsal position obtained after surgery. **a** Day 0, minipig no. 1. **b** Day 0, minipig no 2. **c** Day 0, minipig no. 3. **a**–**c** shows placement of the prosthetic head within the acetabular cup. **d** Lateral X-ray of minipig no. 1, obtained on Day 27, the prosthesis was not completely aligned within the femoral medullary cavity, and new periosteal bone formation was seen (arrow)

The anterior surgical approach resulted in prostheses inserted within the acetabular cup at the postoperative evaluation in all three minipigs (Fig. 4a–c). In the radiograph from minipig No. 2 (Fig. 4b) a fracture line was visible in femur, due to misalignment of the stem during insertion.

### Recovery from surgery: clinical observations

All minipigs were able to stand, eat, and walk with assistance within a few hours after surgery. However, the hip dislocated within a day in minipig no. 2, and after two days in minipig no. 3. Both minipigs had to be euthanized since repositioning the prostheses was impossible. Minipig no. 1 recovered well and was able to walk cautiously on the operated leg, with a moderate lameness, without support a few hours after surgery. Day 1 post-surgery minipig no. 1 was mild to moderately lame but able to walk cautiously around the stable corridor (Video 1). Normal eating and drinking behavior were observed for minipig no. 1; however, an increased resting behavior, i.e., laying and sleeping, was observed. No additional analgesia other than Metacam PO was provided to minipig no. 1 post-surgery. In the week after surgery, the minipig recovered well, the lameness decreased, and nine days post-surgery, the minipig could trot (Video 2). Analgetic Metacam treatment was terminated on day 12 post-surgery. On day 14 post-surgery, the minipig was anaesthetized with propofol, and mid-study x-rays were obtained, and skin sutures removed. The minipig was able to trot and gallop 21 days post-surgery (Video 3). After 27 days minipig no. 1 was euthanized as planned.

### Radiographic evaluation

On days 14 and 27, post-surgery, minipig no. 1 revealed correct placement of the prosthetic head, with good contact with the acetabular cup. However, the prosthetic stem was not completely aligned with the femoral cortex, resulting in the proximal mesh part of the prosthesis being pushed upon the cranial cortex of the femur, where periosteal new bone formation was found (Fig. 4d).

### Gross pathology

In minipig no. 2 and 3 (with prostheses for one and two days, respectively), the prostheses were found to be dislocated dorsocaudally to the acetabulum, with the head of the prosthesis deeply impressed into the musculature, with surrounding hematoma. Edema and hemorrhage were observed in the muscles and subcutis in relation to the surgical wound. The only reaction in the periprosthetic bone was hemorrhage, observed at the distal part of the prosthesis stem.

**Fig. 5:**
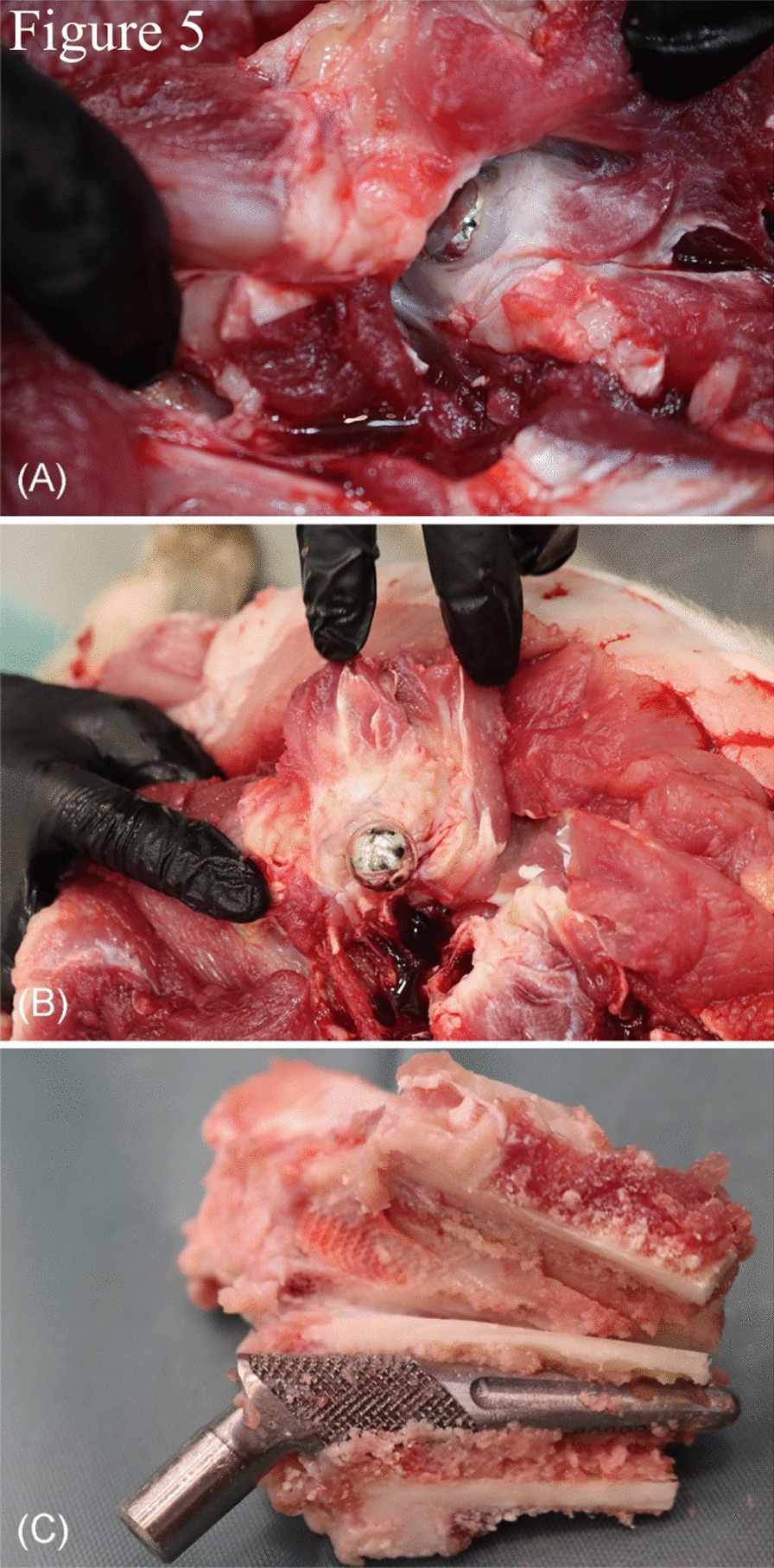
Macroscopic postmortem evaluation of minipig no. 1. **a**, **b** Periarticular fibrosis was covering the prosthesis head and neck. **c** Sagittal sectioning of the bone was necessary to enable prosthesis removal. A fibrotic interface membrane was formed between the prosthesis and the bone

In minipig no. 1, the surgical wound had almost completely healed. The head of the prosthesis was firmly situated in the acetabulum, surrounded by periarticular fibrosis measuring up to 1 cm in thickness. This fibrosis began at the neck of the prosthesis/femur and completely covered the prosthesis head (Fig. 5a, b). The prosthesis was firmly attached to the bone and could not be removed manually. The bone was sectioned sagittal around the prosthesis to allow its removal. A fibrotic interface membrane was present between the bone and the prosthesis (Fig. 5c), while new periosteal bone had formed at the collum. The synovial fluid from the right hip joint was mixed with blood and had a decreased viscosity, while the synovial fluid from the left hip joint showed normal viscosity.

### Histology

In minipig no. 1, granulomatous foreign body reactions related to sutures were present in the cutis, joint capsule, and synovial membrane from the right hip joint. Furthermore, the synoviocytes were necrotic and covered with erythrocytes, fibrin, and a few neutrophils. In the right femur, a layer of connective tissue, necrotic bone fragments, and cell debris was covering the inside of the femoral medullary cavity, forming a narrow fibrotic interface between the bone and the prosthesis. Newly formed trabeculae bone tissue was present at the distal part of the prosthetic stem. In the right acetabulum, erosive arthrosis with cartilage necrosis was present. No muscle lesions were found in relation to the surgical site.

In minipig no. 2, only acute lesions in the form of hemorrhage, edema, fibrin, and a few neutrophils were present in the muscles, synovial membrane, and femur of the right hind leg. Small necrotic bone fragments were also present in the femur and on the surface of the synovial membrane. In the right acetabular cup, an erosive arthrosis was present.

**Fig. 6:**
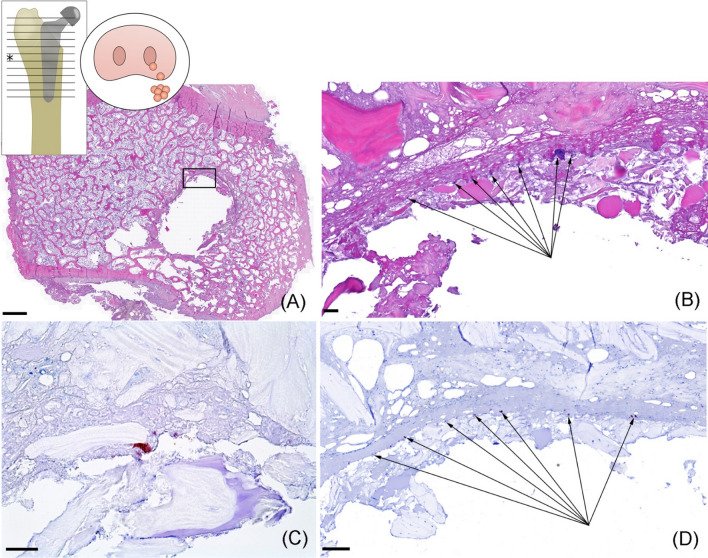
Histology and immunohistochemistry (IHC) of minipig no. 3. Bacteria isolated from the nose prior to surgery was found postmortem inside the bone tissue located within the interface formed between the prosthesis and the bone. All histological sections are from bone slice no. 7 on the insert drawing (*). **a** Overview, inserted black square is zoomed in on (**b**), HE, scalebar = 2 mm. **b** Multiple coccoid bacterial colonies (arrows) located inside the interface between prosthesis and bone, HE, scalebar = 50 µm. **c**, **d** sections of IHC staining towards *staphylococci*. Conformation of IHC red positive *staphylococci* without any surrounding immune cells, **c** scalebar = 25 µm, **d** scalebar = 100 µm

In minipig no. 3, edema, fibrin, and massive infiltration with neutrophils were present in the synovial membrane from the right hip joint. Necrotic bone fragments were found on the surface of the synovial membrane. Additionally, there were focal areas of necrotic synoviocytes and erosive arthrosis in the right acetabular cup. The right femur showed a layer of fibrin, necrotic bone fragments, and cellular debris inside the femoral medullary cavity, forming the interface. Multiple colonies (< 100) of coccoid bacteria were found within the interface, but without any surrounding inflammatory cells observed (Fig. 6). The bacterial colonies stained IHC positive for staphylococci. No lesions were found in the lung, liver, kidney, lymph nodes, left femur, left acetabulum, and left synovial membrane in any of the minipigs.

### Microbiology

All blood samples and cutis obtained during surgery, as well as blood, cutis, subcutis, muscle, and lung samples obtained post-mortem, were sterile. Nose swabs obtained prior to surgery revealed cultures of *Staphylococcus chromogenes* from minipig no. 3. Additionally, pure cultures of high numbers of *Staphylococcus chromogenes* were found in the joint and two of the three bone samples, as well as on the prosthesis from minipig no. 3 (Table 1).

**Table 1:** Microbiological culture results

Minipig no	Euthanized days after surgery	Nose swab prior to surgery	Sutures joint capsule	Synovial membrane	Bone top 1	Bone top 2	Bone bottom	Prostheses
1	27	Mixed culture *	Sterile	Sterile	160 CFU/g—mixed culture dominated by *Staphylo-coccus warneri*	250 CFU/g—mixed culture dominated by *Staphylococcus warneri*	100 CFU/g—mixed culture dominated by *Staphylo-coccus warneri*	Head: 9.33 × 10^1^ CFU/ml *Psychro-bactor immobilis* Stem: 6.8 × 10^2^ CFU/ml mixture of dominating: *Psychrobactor immobilis* and few *Staphylo-coccus eqorum*
2	1	Hemolytic staphylococci *	NS	NS	Sterile	NS	NS	2.27 × 10^2^ CFU/ml *Staphyl-ococcus chromo-genes*
3	2	*Staphylo-coccus chromogenes*	1.6 × 10^4^ CFU/g *Staphylo-coccus chromo-genes*	1.6 × 10^5^ CFU/g *Staphylo-coccus chromo-genes*	1.0 × 10^4^ CFU/g *Staphylo-coccus chromogenes*	1.8 × 10^4^ CFU/g *Acinetobactor woffii* and 2.7 × 10^4^ CFU/g *Streptococcus parauberis*	1.6 × 10^5^ CFU/g *Staphylo-coccus chromo-genes*	6.68 × 10^5^ CFU/ml *Staphylo-coccus chromogenes*

*Not further identified, *NS* not sampled

*Staphylococcus chromogenes* was also isolated in low numbers from the prosthesis of minipig no. 2. Mixed cultures of low numbers were found in the bone and on the prosthesis of minipig no. 1 (Table 1). All *Staphylococcus chromogenes* isolates from minipig no. 3 i.e., nose, bone interface, and prosthesis were found to be identical by whole genome sequencing.

### Synovial fluid evaluation

Hip dislocation in minipig no. 2 and 3 prevented synovial fluid sampling. The cytological examination revealed no signs of an inflammatory reaction in either of the two hip joints from minipig no. 1.

## Discussion

Clinically reliable large animal models are needed to study PJI in its entity. The present study describes the first hip hemiarthroplasty in a Göttingen Minipig, a small pig bred for experimental purposes and used worldwide [19]. The present paper provides a detailed description of the surgical access to the hip joint, the instruments and insertion techniques used, the animal handling, the well-fare protocol, and the post-mortem sampling. The results from this study will facilitate the future of fully functional prosthetic joint infection models in Göttingen minipigs or other minipig breeds. Hip replacement has previously been reported in a minipig model based on Bama minipigs [20], although only focusing on material design and with no detailed description of surgery, handling, or post-mortem sampling [20]. The present study is descriptive in its nature. However, due to the comprehensive protocols and reported experiences it enables best practice in further studies of pig PJI modeling, and it allows for reproducibility in other laboratories or setting. Animal experiments should always be appropriately designed, correctly analyzed, and not at least clearly described [21, 22]. However, it has been documented recently that there is a lack of detail in the way research using animal models is reported, especially also in orthopedics, and the consequence is a lack of reproducibility and more use of animals [21, 22]. Therefore, detailed descriptive studies, as reported in here, adds in to improve 3R in the long run, i.e., refinement and reduction of animal models.

Pigs and small ruminants have been increasingly used in bone infection research in recent decades, as they are the preferred species for large animal models. However, only few arthroplasty studies on pigs exist. In contrast, numerous sheep models of total hip arthroplasties [23–28] and hip hemiarthroplasties [28–30] have been refined and developed. These sheep models initially faced complications such as infection [28] and fractures [23, 25, 26, 32]. So far, sheep arthroplasty models have been used in studies of material design, osseointegration, and surgical techniques [20, 23, 25–32]. Thus, no ovine hip PJI model has been developed [33]. Traditionally, sheep have often been preferred over pigs due to their long bones and their adult size (35–100 kg depending on breed [31]), which make handling easier compared to adult slaughter pigs, which easily reach an adult size of 200–300 kg [34]. However, with the minipigs overcoming size constraints, the pig now represents a highly relevant model for studying human prosthetic joint infections. This is due to their bone structure, anatomy [35, 36], and immune system [37], which are more similar to humans than those of sheep. While minipigs have smaller bones than sheep, they still enable the application of humane or veterinary orthopedic techniques and allow for multiple sampling comparable to clinical practices [38], although a major challenge are the recruitment of large animal facilities. Both ovine and porcine models of implant associated osteomyelitis have successfully been based on intraosseous inoculation of *S. aureus* bacteria, which also is one of the major causes of PJI [4, 15, 16, 33]. Therefore, it seems highly reasonable to develop a future *S. aureus* based PJI minipig model. Although, it should also be acknowledged that PJI are caused by many bacterial species like cutibacterium, enterococcus, and streptococcus species none of which have been examined in porcine bone infection models [4].

**Fig. 7:**
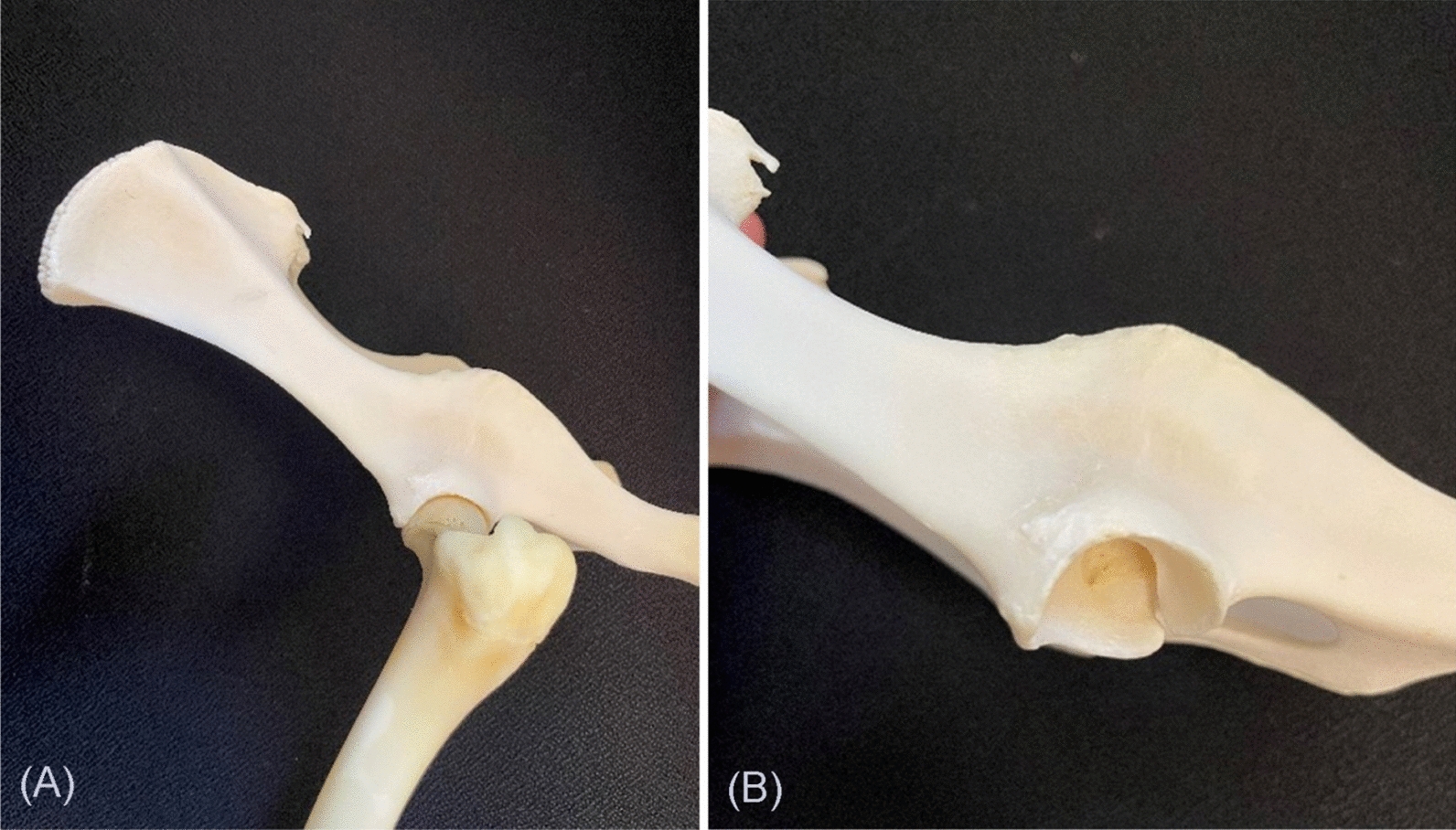
Bones from an adult female Göttingen minipig. Showing the anatomy of the acetabular cup, which is quite shallow and does not encircle the femoral head completely

The present study identified several points of awareness when performing a hip hemiarthroplasty in minipigs. First, there is a risk of caudodorsal dislocation despite postoperative confirmation of the suitability of the acetabular cup's conformation around the hip hemiarthroplasty, resulting in dislocation of the prosthesis in two out of three minipigs in the present study. This may be due to the flat acetabular cup anatomy of Göttingen minipigs, which does not naturally encircle the entire femoral head, relying on stability from the femoral head ligament (Fig. 7).

The following points are important to prevent dislocation: (1) If the prosthesis's angulation is excessively retrograde (retroversion), the risk of the prosthetic head slipping over the caudal acetabular brim is increased. (2) Cleaning the acetabular cup with complete removal of the femoral head ligament is crucial to secure adequate space in the acetabular cup and ensure optimal head-to-acetabulum contact. (3) The early recovery phase after surgery appears to be critical. Since the pig will try to stand as soon as it awakens from anesthesia, even though balance and limb coordination is still considerably affected. This could be avoided by additional assistance during the recovery from anesthesia, i.e., slings [26, 32, 39]. However, training before the surgery is required to make the minipigs accept the slings [40]. Another way to prevent dislocations would be to insert a prosthetic acetabular cup to secure better containment of the joint. This approach has previously been successful [20]. However, inserting an acetabular component will extend the surgical time. If an acetabular cup could diminish the risk of dislocations and eliminate the cartilage damage, this would be advisable.

Despite meticulous efforts to maintain strict aseptic surgery and necropsy, bacteria were detected post-mortem within the minipigs. Mixed cultures and low bacterial numbers are likely attributed to contamination during surgery or sampling at necropsy and should be mitigated by improved sterile efforts. In contrast, the presence of high numbers of pure cultures of *Staphylococcus chromogenes* in samples obtained from the joint, bone and prosthesis, along with the consistent findings of coccoid IHC-positive bacteria in the histological bone sections, strongly suggests the development of a genuine PJI in minipig no. 3. *Staphylococcus chromogenes* is a commensal of the skin and mucosal flora in pigs [41]. Furthermore, whole-genome sequencing revealed that the *Staphylococcus chromogenes* isolates from the nose swab, the bone, and the prosthesis were identical. Although not intentionally induced, the occurrence of a natural PJI in minipig no. 3 confirms the suitability of the hip arthroplasty minipig model for future experimental modeling of PJI, even with a very low and thus clinically realistic dose of inoculated bacteria. An airborne bacterial pathogenesis is likely and mitigation by better ventilation of the surgical theater could be considered. An interesting observation was the presence of multiple bacterial colonies at the bone-prosthesis interface seen in the histological bone sections. These colonies appeared to be undetected by the immune system, as they were not surrounded by neutrophils or other immune cells (Fig. 6). Known from pig studies of sepsis [42] and aseptic tissue damage [43], neutrophils are recruited to the site of injury or infection within hours. However, in this study, not a single neutrophil was observed at the infected bone-prosthesis interface 48 h after surgery, presumed to be the time of bacterial inoculation. It could be speculated that this is caused by the changed microenvironment due to tissue damage secondary to prosthesis insertion, disrupting the blood supply at the bone-prosthesis interface, thereby preventing the recruitment and migration of neutrophils and other immune cells. Consequently, the bacteria are left alone and unhindered to multiply and colonize the implant surface, and in the end, creating a recalcitrant biofilm infection [44]. This finding can contribute to understanding why prostheses are notably vulnerable to bacterial colonization and emphasizes the necessity of prompt actions to eradicate bacteria immediately after prosthesis insertion.

Despite being a descriptive study, several limitations were identified. In summary, one limitation was the small sample size of only three minipigs, which all were euthanized at different time-points. Furthermore, the study identified a high luxation rate which was identified to be related to the porcine pelvis anatomy and post-surgical animal behavior. Therefore, to avoid luxation and thus induce reproducibility in future models the acetabular cup component must be included. An alternative is a custom-made prosthesis design which, however, would be extremely expensive. A commercial veterinary prosthesis allows for a standardized procedure across global laboratory settings. In future PJI research, based on the present protocols, bacterial inoculation should be performed during the surgical procedure to reflect the most common pathogenesis of PJI. A low volume of inoculum, i.e. 10 microL as previously reported in a porcine model of osteomyelitis, should be used to secure correct inoculum placement along the prosthesis, just before the final press-fit steps.

## Conclusion

This study provides a detailed description of the surgical technique used to perform hip hemiarthroplasty in the Göttingen minipig, along with the post-mortem sampling procedure. Such technical descriptions are indispensable for the future development of hip PJI models in minipigs, which will be of significant clinical relevance for advancing PJI treatment. Additionally, the occurrence of spontaneous PJI in one minipig validates the suitability for infection development and provides novel insights into the early stages of infection, i.e., delayed immune cell recruitment. The study was limited by few animals, and an identified high risk of luxation. Further research to model PJI should, therefore, include an acetabular cup and experimental bacterial inoculation.

## Supplementary Information

Supplementary file 1. 

Supplementary file 2. 

Supplementary file 3. 

Supplementary file 4. 

Supplementary file 5. 

## Data Availability

No datasets were generated or analysed during the current study.

## Abbreviations

PJI: Prosthetic joint infection
HE: Hematoxylin & Eosin
IHC: Immunohistochemistry
L6: Lumbar vertebrae 6
S1: Sacral vertebrae 1
