# Supplementary material for: First hip hemiarthroplasty in a Göttingen Minipig; surgical and post-mortem protocol
Source: J Orthop Surg Res. 2024 Sep 6;19:549. doi: 10.1186/s13018-024-05040-z (PMC11380332; doi:10.1186/s13018-024-05040-z)
Supplement: Supplementary file 1 [file 13018_2024_5040_MOESM1_ESM.docx]

# **First Hip Hemiarthroplasty in Göttingen Minipigs; Surgical and Post-mortem Protocol**

## Supplementary - S1

**Anesthesia, analgesia and euthanization protocol**

The minipigs were sedated by an intramuscular injection of 0.08 ml/kg body weight (BW) with Zoletil Mixture consisting of Zoletil 50 Vet. dry matter: 125 mg tiletamine + 125 mg zolazepam (Zoletil® 50 Vet. (50 mg/ml); Virbac) dissolved with 6.25 ml xylazin (Xysol Vet. (20 mg/ml); Scanvet), 1.25 ml ketamine (Ketaminol Vet. (100 mg/ml), MSD Animal Health A/S), 2.0 ml butorphanol (Torbugesic (10 mg/ml); Zoetis Animal Health ApS) and 2.0 ml metadon (Comfortan Vet. (10 mg/ml); Dechra Veterinary Products). Sedation was achieved approximately 10 to 15 minutes following injection. Anesthesia was maintained by intravenous infusion of Propofol 5 mg/kg BW/h (Propofol (10 mg/ml); B. Braun). Intraoperative analgesia was achieved by intravenous infusion of Fentanyl 0.75 mg/kg BW/ hr. (Fentanyl (50 µg/ml); Hameln). Fentanyl administration was initiated 15 minutes prior to surgery. Intraoperative and postoperative analgesia was achieved with epidural block, consisting of Morfin 0.1 mg/kg (Morfin Dak (20 mg/ml)), Bupivacain 0.3 mg/kg (Marcain Spinal (5 mg/ml)) and sterile isotonic saline, in a total volume of 6 ml, sterilely injected to the epidural space between the lumbosacral junction of L6 and S1 (Figure 1) [9], 15 minutes prior to surgery. The postoperative analgesic time for this procedure is reported to be up to 24 hours in dogs [10]. Prior to surgery the minipigs received an intramuscular injection of Meloxicam 0.4 mg/kg (Metacam (20 mg/ml), [Boehringer Ingelheim Animal Health Nordics A/S](https://medicintildyr.dk/company/Boehringer-Ingelheim)), providing postoperative analgesia for 24 hours.

Following surgery, the minipigs received daily oral analgetic treatment with Meloxicam 0.4 mg/kg (Metacam (15 mg/ml); Boehringer Ingelheim Animal Health Nordics A/S). In case of lameness or observed pain behavior of the minipigs, intramuscular injection of Buprenorphine 0.1 ml/kg (Bupaq Vet. Multidose (0.3 mg/ml), Salfarm Denmark A/S), was provided every eighth hour. At euthanization, the minipigs were sedated by intramuscular injection of Zoletil Mixture 0.12 ml/kg followed by an intravenous injection of an overdose of pentobarbital 0.35 ml/kg (Euthanimal (400 mg/ml), Alfasan). Ringers Acetat 10 ml/kg BW/h was administered during surgery, and a urinary catheter was placed.
