# Supplementary material for: First hip hemiarthroplasty in a Göttingen Minipig; surgical and post-mortem protocol
Source: J Orthop Surg Res. 2024 Sep 6;19:549. doi: 10.1186/s13018-024-05040-z (PMC11380332; doi:10.1186/s13018-024-05040-z)
Supplement: Supplementary file 2 [file 13018_2024_5040_MOESM2_ESM.docx]

| Parameter | day: | day: | day: | day: | day: | day: | day: | day: |
| --- | --- | --- | --- | --- | --- | --- | --- | --- |
| Date, time, initials |  |  |  |  |  |  |  |  |
| Notes: |  |  |  |  |  |  |  |  |
| **1. Interest in the surroundings**  - Normal  - Moderate  - No interest |    |    |    |    |    |    |    |    |
| **2. Activity level**  - Normal active behavior  - Passive behavior  - Hyperactive/ manic behavior |    |    |    |    |    |    |    |    |
| **3. Vocalization**  - Normal grunts  - None  - Spontaneous vocalization  - Handling induced vocalization |      |      |      |      |      |      |      |      |
| **4. Feeding**  - Normal/ eating all feed  - Reduced diet consumption  - Not eating |    |    |    |    |    |    |    |    |
| **5. Fecal score**  - None  - Normal  - Soft  - Dry |      |      |      |      |      |      |      |      |
| **6. Respiration**  - Normal  - Superficial  - Deep  - Abnormal sounds |     |     |     |     |     |     |     |     |
| **7. Posture**  - Normal  - Abnormal head posture  - Abnormal bag posture  - Abnormal leg posture |     |     |     |     |     |     |     |     |
| **8. Tail position**  - Normal position and movement  - Anormal movement  - Tail between hind legs  - Tail pointing upwards |     |     |     |     |     |     |     |     |
| **9. Eyes**  - Normal open eyes  - Partly open eyes - Closed eyes |    |    |    |    |    |    |    |    |
| **10. Ears**  **-** Pointing forward and attentive  - Pointing backwards  - Laying down the neck |    |    |    |    |    |    |    |    |
| **10. Pain-related behaviour***  - Observed  - Not observed |   |   |   |   |   |   |   |   |
| **11. Lameness****  - None  - Mild lameness  - Moderate lameness  - Severe lameness  - Not using/ supporting on the leg |       |       |       |       |       |       |       |       |
| **12. Getting up without trouble**  - Yes  - No  - Was already standing |    |    |    |    |    |    |    |    |
| **13. Wound**  - Normal and dry  - Redness  - Swelling  - Drainage  - Separation of wound edges |       |       |       |       |       |       |       |       |

*a) teeth grinding, b) quivering, c) abdominal press, d) stiff/ impaired movements, e) breathing hard, f) changed facial expression (ear backwards, angled eyes, wrinkled nose) **What leg
